# Methicillin Resistant Staphylococci Isolated from Goats and Their Farm Environments in Saudi Arabia Genotypically Linked to Known Human Clinical Isolates: a Pilot Study

**DOI:** 10.1128/spectrum.00387-22

**Published:** 2022-08-01

**Authors:** Wael El-Deeb, Rory Cave, Mahmoud Fayez, Naser Alhumam, Sayed Quadri, Hermine V. Mkrtchyan

**Affiliations:** a Department of Clinical Sciences, College of Veterinary Medicine, King Faisal University, Al-Hofuf, Al-Ahsa, Saudi Arabia; b Department of Internal Medicine, Infectious Diseases and Fish Diseases, Faculty of Veterinary Medicine, Mansoura University, Mansoura, Egypt; c School of Biomedical Sciences, University of West London, London, United Kingdom; d Al Ahsa Veterinary Diagnostic Laboratory, Ministry of Environment, Water and Agriculture, Al-Hofuf, Al-Ahsa, Saudi Arabia; e Veterinary Serum and Vaccine Research Institute, Ministry of Agriculture, Cairo, Egypt; f Department of Microbiology and parasitology, College of Veterinary Medicine, King Faisal University, Al-Hofuf, Al-Ahsa, Saudi Arabia; g Division of Microbiology and Immunology, Department of Biomedical Sciences, College of Medicine, King Faisal University, Al-Hofuf, Al-Ahsa, Kingdom of Saudi Arabia; University of Calgary

**Keywords:** MRS, MRSA, MRSE, Saudi Arabia, environment, genotypes, goat

## Abstract

We conducted a pilot whole genome sequencing (WGS) study to characterize the genotypes of nine methicillin resistant staphylococci (MRS) isolates recovered from goats and their farm environments in Eastern Province, Saudi Arabia, between November 2019 to August 2020. Seven out of nine isolates were methicillin resistant Staphylococcus aureus (MRSA), and two were methicillin resistant Staphylococcus epidermidis (MRSE). All MRSA isolates possessed genotypes previously identified to infect humans, including isolates harboring ST6-SCC*mec* IV-t304 (*n* = 4), ST5-SCC*mec* VI- t688 (*n* = 2) and ST5-SCC*mec* V-t311 (*n* = 1). 2 MRSA isolates possessed plasmids that were genetically similar to those identified in S. aureus isolates recovered from humans and poultry. In contrast, plasmids found in three MRSA isolates and one MRSE isolate were genetically similar to those recovered from humans. All MRSA isolates harbored the host innate modulate genes *sak* and *scn* previously associated with human infections. The genotypes of MRSE isolates were determined as ST35, a well-known zoonotic sequence type and ST153, which has been associated with humans. However, the MRSE isolates were untypeable due to extra *ccr* complexes identified in their SCC*mec* elements. Moreover, we identified in ST153 isolate SCC*mec* element also harbored the Arginine Catabolic Mobile Element (ACME) IV. All MRS isolates were phenotypically resistant to trimethoprim-sulfamethoxazole, an antibiotic for the decolonization of MRS. Three isolates carried antibiotic resistance genes in their SCC*mec* elements that were not previously described, including those encoding fusidic acid resistance (*fusC*) and trimethoprim resistance (*dfrC*) incorporated in the MRSA SCC*mec* VI.

**IMPORTANCE** Our findings demonstrate a possible cross-transmission of methicillin resistant staphylococci between goats and their local environments and between goats and humans. Due to ever increasing resistance to multiple antibiotics, the burden of MRS has a significant impact on livestock farming, public health, and the economy worldwide. This study highlights that implementing a holistic approach to whole genome sequencing surveillance in livestock and farm environments would aid our understanding of the transmission of methicillin resistant staphylococci and, most importantly, allow us to implement appropriate infection control and hygiene practices.

## INTRODUCTION

Methicillin resistant staphylococci (MRS) have become a major threat to human and animal health due to the infections they cause and have become increasingly challenging to treat as these bacteria developed resistance to multiple antibiotics, predominantly due to the overuse of antibiotics in health care and agriculture settings (1–3). Many staphylococcal species are commensal to animals and humans but can act as opportunistic pathogens (4). Staphylococcus aureus and Staphylococcus epidermidis are common causes of staphylococcal infections in humans and animals and can transmit across host species barriers either via direct contact, the environment, or the food chain (5).

Methicillin resistance is derived from the *mecA* gene conferring resistance to all beta-lactam antibiotics, including carbapenems, cephalosporins, cephamycins, and monobactams. The *mecA* gene is believed to have originated from staphylococcal species of animal origin before successfully transferring across other staphylococci species via the SCC*mec* mobile genetic element (6, 7). The *mecA* gene has widely been associated with methicillin resistant S. aureus (MRSA); one of the leading causes of hospital-acquired infections in the world, as well as a common cause of infections in the community and livestock (3, 8, 9) and methicillin resistant S. epidermidis (MRSE), which, although lacking the arsenal of virulence factors of S. aureus, has been associated with nosocomial and medical device infections as well as with community and livestock infections (10–15).

Goats are common agricultural animals in many Middle Eastern countries and are used as a source of meat and milk. In Saudi Arabia, an estimated 3.7 million heads of goats in 2019 produced31,839 tons of meat and 68,694 tons of milk (16). Mastitis, the infection of the mammary gland, is common among goats and regularly caused by Staphylococcus aureus (17–19). Goat mastitis causes significant economic losses due to the reduced quality and quantity of milk and, in some cases, leads to the slaughter of the animal (20). The economic impact of mastitis in goats is further exacerbated by antibiotic resistance, making it harder to treat. A study published in 2015 reported that among the isolates recovered from the mastitis milk in the farms in Eastern Province, Saudi Arabia, 33.8% were S. aureus, of which 9.2% were MRSA compared to 4.2% and 0.6%, respectively, recovered for normal milk (21). In addition, the authors (21) found that 17.9% of the isolates recovered from nasal swabs of diseased animals were S. aureus, of which 2.6% were MRSA compared to 10.2% and 0.8%, respectively, of nasal swabs recovered from healthy animals. However, they provided no information on whether the genotypes of the MRSA isolates recovered from goats in Saudi Arabia were similar to those commonly found in livestock or associated with humans or the environment. In this study, we provide insights into the genotypes of MRS isolates recovered from goats and their farm environments in Saudi Arabia and analyze the genetic features to identify the possible source of transmission.

## RESULTS

### Speciation and genotyping of MRS isolates from goats and their environment.

In total, 57 staphylococci isolates were recovered from November 2019 to August 2020 from goats and their surrounding environments on a farm in Eastern Province, Saudi Arabia (Table S2). Nine out of 57 (15.7%) isolates that showed resistance to methicillin were sequenced (Table 1). Areas where MRS isolates were recovered from include goat's nasal swabs (*n* = 6), goat’s milk (*n* = 1), goat's drinking water (*n* = 1) and from the soil (*n* = 1). S. epidermidis (*n* = 2) was only isolated from the nasal swabs, whereas S. aureus (*n* = 7) was isolated from the nasal swabs (*n* = 4), goat milk (*n* = 1), drinking water (*n* = 1), and the soil (*n* = 1).

**TABLE 1:** Speciation and genotyping of MRS isolates from goats and the surrounding environments

Isolate no.	ID	Species	Source^*a*^	Isolation date	MLST	CC	Spa type	SCC*mec*
1	SE1	S. epidermidis	GNS	12/2019	153	NA^*b*^	NA	*mec* class B with two *ccr* class 4 and one *ccr* class 2
2	SE2	S. epidermidis	GNS	1/2020	35	NA	NA	VI with an extra *ccr class4*
3	SA1	S. aureus	GM	8/2020	5	5	t311	V
4	SA2	S. aureus	GNS	2/2020	5	5	t688	VI
5	SA3	S. aureus	GNS	1/2020	6	5	t304	IV
6	SA4	S. aureus	DW	1/2020	5	5	t688	VI
7	SA5	S. aureus	GNS	3/2020	6	5	t304	IV
8	SA6	S. aureus	GNS	11/2019	6	5	t304	IV
9	SA7	S. aureus	Soil	11/2019	6	5	t304	IV

a GNS = goat’s nasal swab, GM = goat’s milk, DR = drinking water.

b NA means ‘not applicable’ as *S. epidermidis* does not have spa type of clonal clusters.

Whole genome sequencing analysis showed that all S. aureus isolates belonged to clonal complex (CC) 5. The most common S. aureus genotype isolated was ST6-MRSA-SCC*mec* IV-spa type t304 (ST6-MRSA-SCC*mec* IV-t304) (*n* = 4), which was identified in isolates recovered from nasal swabs (*n* = 3) and the soil (*n* = 1). Two isolates recovered from goat's nasal swab (*n* = 1) and drinking water (*n* = 1) possessed ST5-MRSA-SCC*mec* VI-t688 and one isolate recovered from goat's milk possessed ST5-MRSA-SCC*mec* VI - t311. In addition, S. epidermidis isolates belonged to ST153-MRSE-*mec* class B, with two *ccr* class 4 (*ccrA4/B4* complex) and one ccr class 2 (*ccrA2/B2* complex) (*n* = 1) and ST35-MRSE-SCC*mec* VI with an extra *ccr* class 4 (*n* = 1).

### Determining the genetic similarities of MRS isolates recovered from goats and their environments.

**FIG 1:**
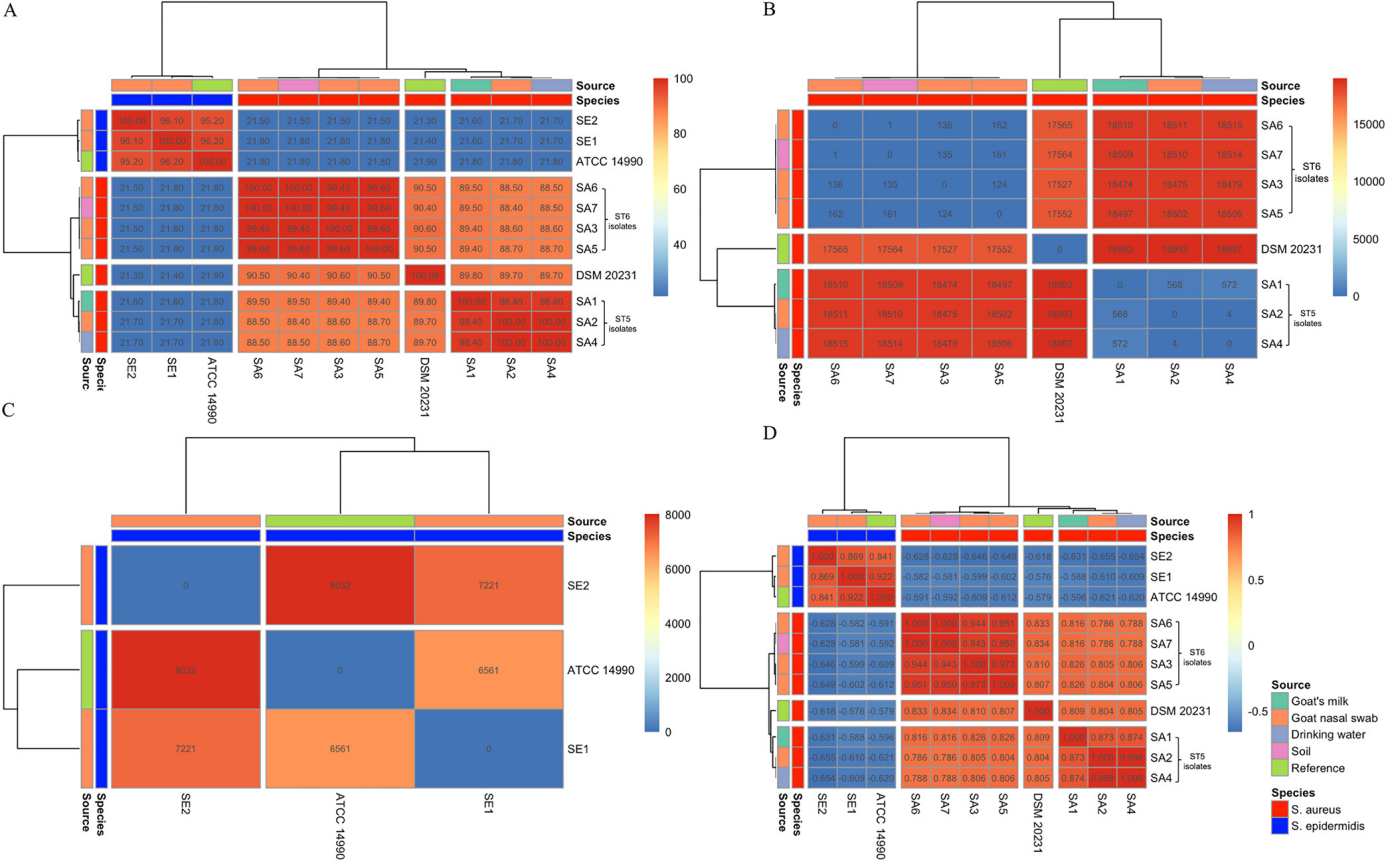
Hierarchy cluster of MRS isolates from goats and farm environments in Eastern Province, Saudi Arabia, showing genomic similarities between S. aureus isolates from different sources. (A) dDDH. (B) Pairwise SNP distance of the core genome containing 2,492,080 nucleotides for S. aureus isolates. (C) Pairwise SNP difference of the core genome containing 2,232,743 nucleotides for S. epidermidis isolates. (D) Gene correlation between isolates.

To determine genetic similarities of MRS isolates recovered from goats and their surrounding environments, we performed digital DNA-DNA hybridization (dDDH), pairwise SNP distance of the core genome, and correlation between shared genes within the isolate's genome (Fig. 1). We found that the isolates cluster based on their species and MLST genotypes using all four methodologies. The dDDH scores of the MRSA isolates belonging to the same sequence type that has been recovered from goats and their surrounding environments were high (MRSA ST5 isolates dDDH range from 98.40 to 100% and MRSA ST6 isolates dDDH range from 99.4 to 100%) with small SNP differences (MRSA ST5 isolates SNP difference range 572-4 and MRSA ST6 isolates SNP difference range 162-1) and high correlation of genes (MRSA ST5 isolates gene correlation range *r* = 0.873-0.998 and MRSA ST6 isolates correlation range *r* = 0.950-1.0). Interestingly, we found that SA7 isolated from soil and SA6 from goat's nasal swab isolated at the same time (November 2019) were genetically identical by dDDH (dDDH = 100%), with a pairwise SNP distance of one and a correlation of *r* = 1 for genes they share. The SNP difference between SA7 and SA6 was a synonymous mutation within the Oxygen-dependent choline dehydrogenase gene *betA*. In addition, SA2 recovered from a goat nasal swab and SA4 from drinking water that were isolated 1 month apart (January 2020 and February 2020, respectively) were also shown to be genetically identical by dDDH (dDDH = 100%) with a pairwise SNP distance of 4 and a gene correlation of *r* = 0.998. Moreover, SA2 possessed four genes that had not previously been characterized and were not present in the SA2 genome and SA4 possessed one gene that had not been characterized and was not present in the SA2 genome.

### Antibiotic resistance genotyping and phenotyping.

**FIG 2:**
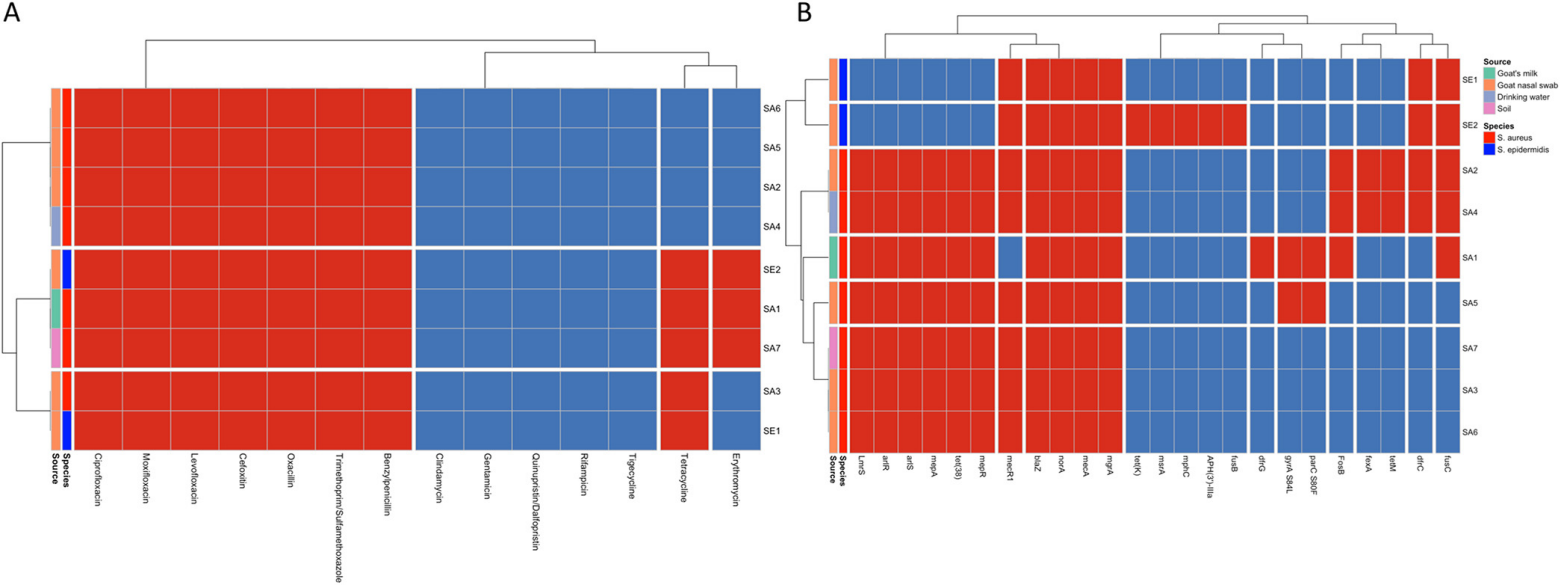
Hierarchy cluster heatmap of MRS isolates from goats and surrounding farm environments in Eastern Province, Saudi Arabia. (A) Antibiotic resistance phenotype profiles. (B) Genotype resistance phenotypes. Red tiles presence of antibiotic resistance genotype/phenotype; blue tiles absence of antibiotic resistance phenotype/genotype.

All MRS isolates recovered from goats and surrounding environments tested against a panel of 14 antibiotics were resistant to benzylpenicillin, oxacillin and cefoxitin (all penicillin class of antibiotics); levofloxacin, moxifloxacin, and ciprofloxacin (all fluoroquinolones class of antibiotics) and trimethoprim-sulfamethoxazole (diaminopyrimidines- sulfonamide class of antibiotic) (Fig. 2A). However, all isolates were susceptible to rifampicin (antimycobacterial class of antibiotic), tigecycline (glycylcyclines class of antibiotic), quinupristin-dalfopristin (streptogramin class of antibiotics), gentamicin (aminoglycoside class of antibiotic), and clindamycin (macrolide class of antibiotic). We found that SA7 and SA6 differed in their resistance profile (SA7 was resistant to erythromycin [macrolide class of antibiotic] and tetracycline [tetracycline class of antibiotic], whereas SA6 was sensitive) despite being nearly genetically identical. All MRS isolates were sensitive to rifampicin, tigecycline, quinupristin-dalfopristin, gentamicin, and clindamycin. We found S. epidermidis isolates were resistant to 9 (SE2) and 8 (SE1), whereas S. aureus isolates were resistant to 9 (*n* = 2: SA1 and SA7) and 7 (*n* = 6: SA2, SA3, SA4, SA5, and SA6) antibiotics.

Antibiotic resistance genotyping (Fig. 2B) showed that all MRS isolates possessed not only the *mecA* gene but also *blaZ* (encoding for beta-lactam resistance) and *norA* genes (encoding for fluoroquinolones resistance) and *mgrA* (genes responsible for regulating *norA*). In addition, *lmrS* (encoding for aminoglycoside, macrolide antibiotic, phenicol, diaminopyrimidine and oxazolidinone resistance), *mepA* and *mepR* (encoding for tetracycline and glycylcycline resistance), *arlR*, and *arlS* (genes responsible for regulating *norA*), and *tet*(38) (encoding for tetracycline resistance) were all present in all sequenced S. aureus isolates but were absent in S. epidermidis isolates. Other antibiotic resistance genes detected were *mecR1* (regulator gene for *mecA*) in S. epidermidis (*n* = 2) and in S. aureus (*n* = 6); *dfrC* (diaminopyrimidine resistance) and *fusC* (fusidic acid resistance) in S. epidermidis (*n* = 2) and in S. aureus isolates (*n* = 3); *fosB* (fosfomycin resistance) in S. aureus isolates (*n* = 3); *fexA* (phenicol resistance) in S. aureus isolates (*n* = 2); *tetM* (tetracycline resistance) in S. aureus isolates (*n* = 2) and S. epidermidis isolates (*n* = 2); *msrA* (streptogramin, macrolide and streptogramin B resistance); *mphC* (macrolide resistance), *fusB* (fusidic acid resistance), *APH(3′)-*IIIa (aminoglycoside resistance), and *tetK* (tetracycline resistance) in an S. epidermidis isolate (*n* = 1), and *dfrG* (diaminopyrimidine resistance) in an S. aureus isolate (*n* = 1). Additionally, we detected antibiotic resistance conferring mutation *parC* S80F and *gyrA* S84L (fluoroquinolone resistances) in S. aureus (*n* = 2). S. epidermidis isolates had 12 (SE2) and 7 (SE1) genes/mutations that encode for antibiotic resistance, whereas S. aureus isolates had 16, (*n* = 2, SA2 and SA4), 15, (*n* = 1, SA1), 13 (*n* = 1, SA5), and 11 (*n* = 3, SA3, SA6, SA7) genes/mutations that encode for antibiotic resistance.

### S. aureus virulence genotyping.

**FIG 3:**
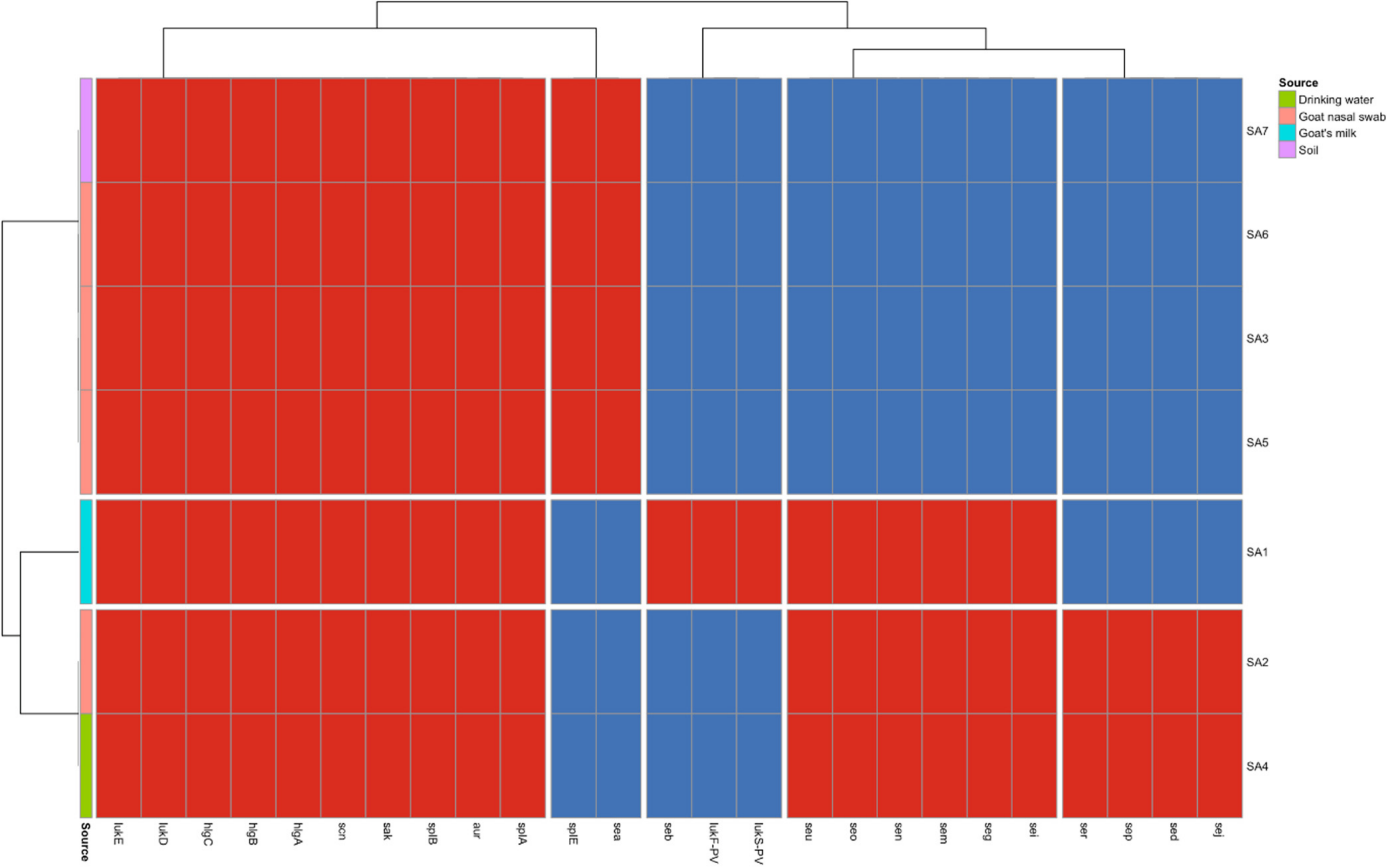
Acquired virulence genes of MRSA isolates recovered from goats and their surrounding farm environments in Eastern Province, Saudi Arabia.

Twenty-five acquired virulence genes were detected in the sequenced MRSA isolates that were recovered from goats and the surrounding environments (Fig. 3). All isolates harbored toxin-producing genes, including *hlgA*, *hlgB*, *hlgC*, *lukD*, and *lukE*; exoenzyme genes *aur*, *splA.* and *splB,* and the host innate modulate genes *sak* and *scn*. Only SA1 isolate recovered from goat’s milk had the Panton-Valentine leucocidin toxin gene *lukF-PV* and *lukS-PV* and the Staphylococcus enterotoxin B gene *seb*. Overall, 20 (*n* = 2), 19 (*n* = 1) and 12 (*n* = 4) acquired virulence genes were detected S. aureus isolates.

### Plasmids found in MRS isolates recovered from goats and the environment.

**FIG 4:**
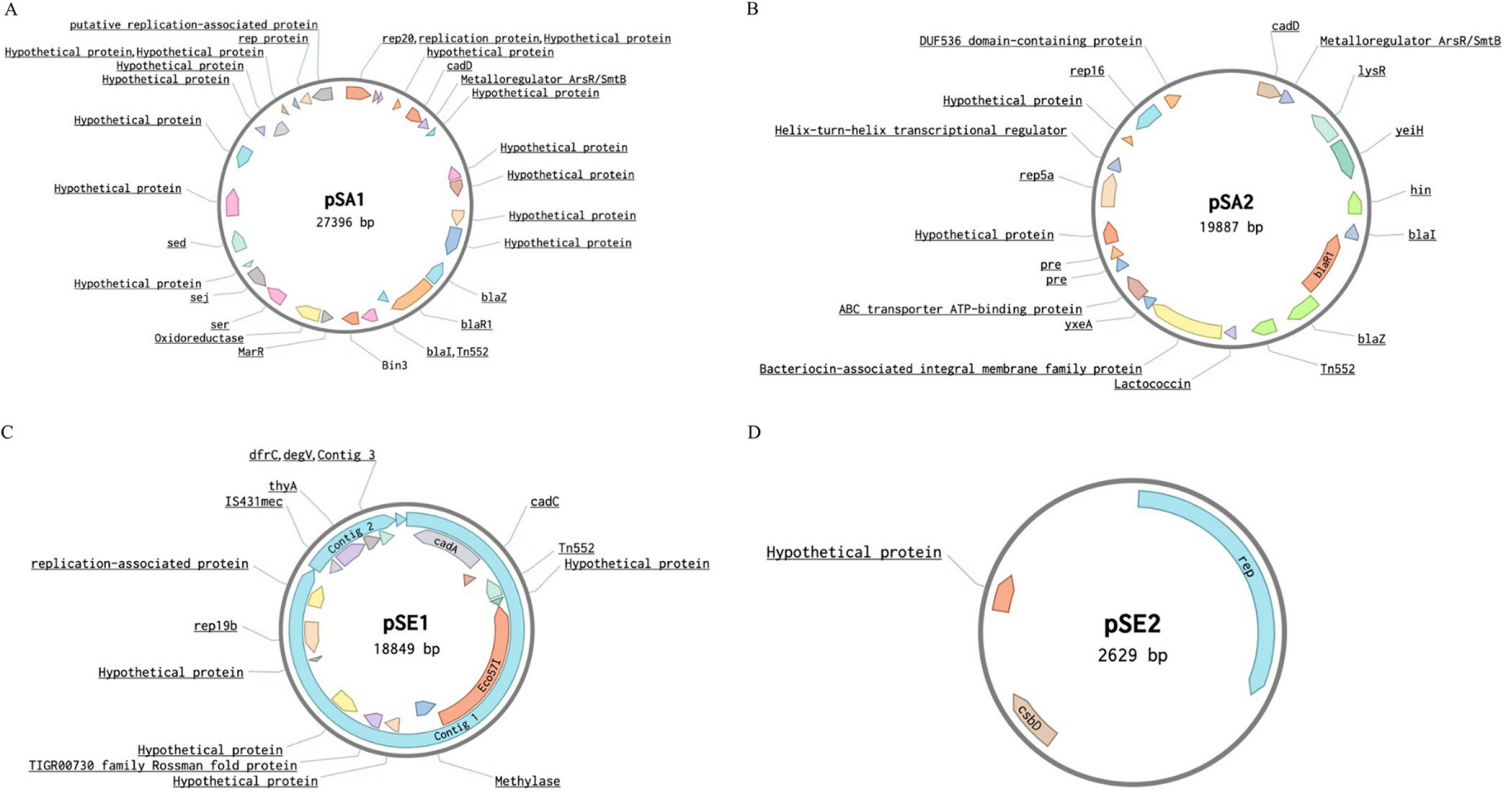
Maps of four plasmids isolated from MRS from goats and their surrounding farm environments in Eastern Province, Saudi Arabia.

All isolates except for SA1 harbored a single plasmid (Fig. 4). Two different plasmids were identified in S. aureus isolates, and two different plasmids were identified in S. epidermidis isolates. Plasmid SA1 (pSA1) was detected in isolates (SA2 and SA4) carrying the ST5-MRSA-SCC*mec* VI -t688 genotype, whereas plasmid SA2 (pSA2) was found in all isolates (SA5, SA6, and SA7) with the ST5-MRSA-SCC*mec* VI -t688 genotype. Plasmid SE1 (pSE1) was identified in isolate SE1 and plasmid SE2 (pSE2) was identified in an isolate SE2 only.

Plasmid pSA1 and pSA2 (Fig. 4A and B) shared the Tn552 transposon; the beta-lactamase gene operon (*blaZ*, *blaI*, and *blaR*), cadmium metal resistance gene *cadD*, along with the metalloregulatory transcriptional repressors *ArsR/SmtB*. The only gene that pSE1 (Fig. 4C) shared with pSA1 and pSA2 was the Tn552 transposon. However, pSE did contain *cadA* and *cadC* genes responsible for cadmium resistance. We found that pSE2 did not share any genes with the other plasmids and that overall, the plasmid only contained three genes, of which one was hypothetical protein, the other two being the replication initiation protein and the general bacterial stress response protein *csbD*. Genes unique to pSA1 included the *rep20* plasmid replication gene, three endotoxin genes (*sed*, *ser,* and *sej)*, the *marR* family transcriptional regulator, putative replication-associate protein, recombinase gene *binIII* and the oxidoreductase gene. Genes that were unique to pSA2 were the two plasmid replication genes (*rep16* and *rep5a*) and two recombinase genes, DNA invertase (*hin*), the *lysR* transcription factor, a helix-turn-helix transcriptional regulator gene and *Lactococcin* bacteriocin gene, the *yxeA* gene, which has not been charactered and the ABC transporter ATP binding protein. Genes unique to pSE1 were the *rep19a* plasmid replicon gene, the restriction-modification methylase *Eco57I* gene, a methylase gene, TIGR00730 family Rossman fold protein gene; the insertion sequence IS431*mec*, *thyA* gene involved in the biosynthesis of thymidylate, *dfrC* gene responsible for trimethoprim resistance, and the *degV* gene that binds long-chain fatty acids.

**FIG 5:**
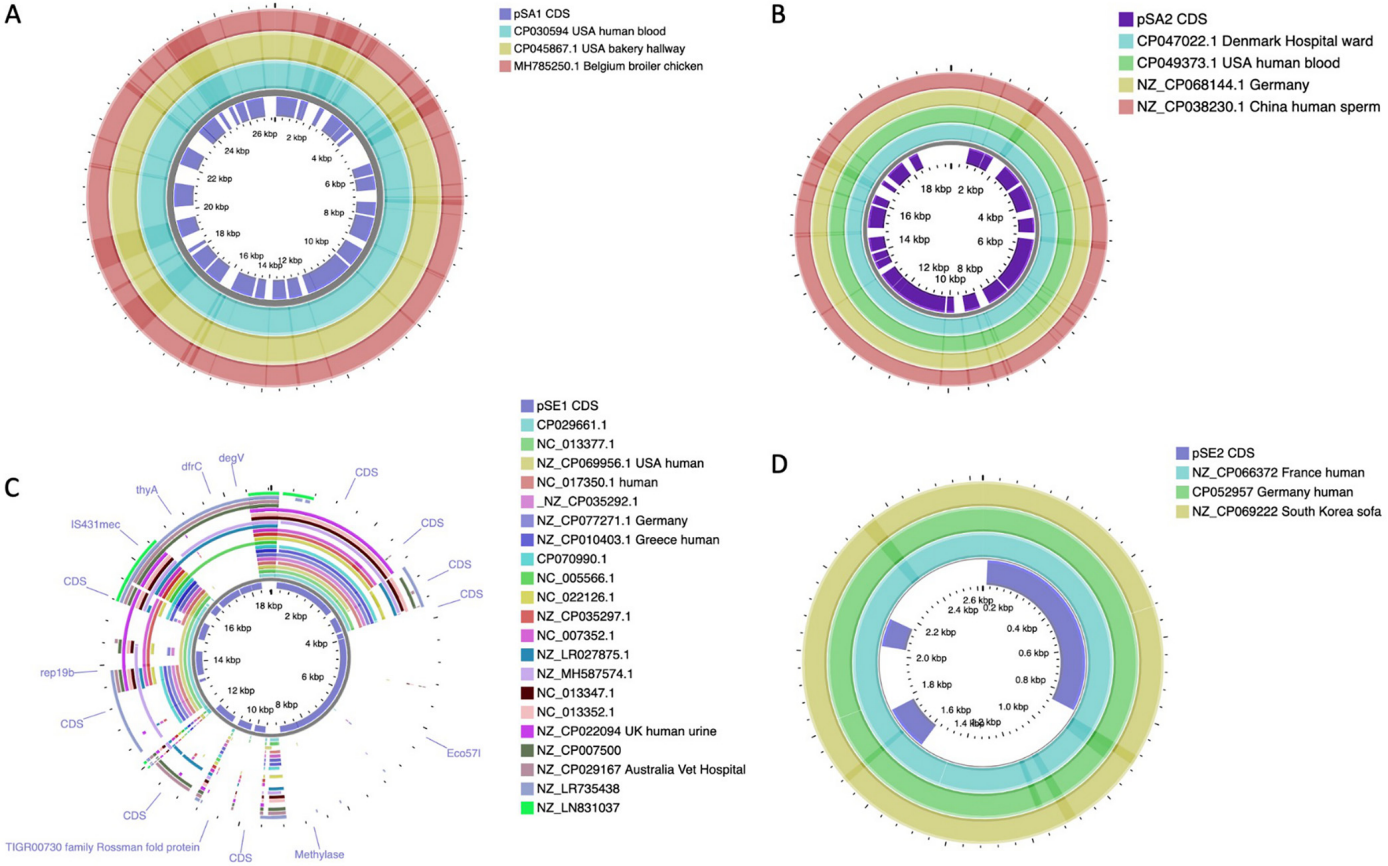
BLAST analysis of plasmids that had hits from MASH distance analysis showing pSA1, pSA2 and pSA3 were not novel plasmids; however, pSE1 plasmid appeared to be novel due to low similarities to other plasmids on the database. (A) pSA1, (B) pSA2, (C) pSE1, and (D) pSE2.

To investigate the novelty of plasmids found in our MRS isolates recovered from goats and the surrounding environment, we used PLSDB mash distance analysis (Tables S3, S4, S5 and S6) and blast (Fig. 5). We found that the plasmids found in MRSA isolates in this study have previously been reported in isolates recovered in different countries and sources. Plasmid pSA1 had 73 isolates with shared hashes of >900 (Table S3). Most of these plasmids were found in S. aureus isolates recovered from humans, including clinical samples (e.g., blood) in the USA isolated (accession no: CP030594); however, we did find pSA1 had hits with S. aureus isolates recovered from broiler chickens in Belgium (accession no: MH785250) and a bakery environment in the USA (accession no: CP045867) (Fig. 5A). Plasmid pSA2 had four hits with shared hashes of >900 with S. aureus isolates recovered from isolates from human and hospital wards in Denmark (accession no: CP047022), the USA (accession no: CP049373) and China (Fig. 5B) (Table S4). We also found that pSE2 from MRSE isolate SE2 had three hits with shared hashes >900 with S. epidermidis isolates recovered from France, Germany, and South Korea (Fig. 5D) (Table S6). Two of the isolates were recovered from humans (accession no: CP066372 and CP052957), whereas another isolated was recovered from a sofa in South Korea (accession no: CP069222). In addition, there were no isolates on the database with shared hashes >900 with the pSE1 found in MRSE SE1 in this study (Table S5). All 23 isolates which had hits for pSE1 were used for blast comparison to determine the genes they shared (Fig. 5C). We found that none of the plasmids shared the *Eco57I*, methylase gene, or the TIGR00730 family Rossman fold protein gene, along with three uncharacterized genes.

### SCC*mec* structure of MRS isolates from public settings.

**FIG 6:**
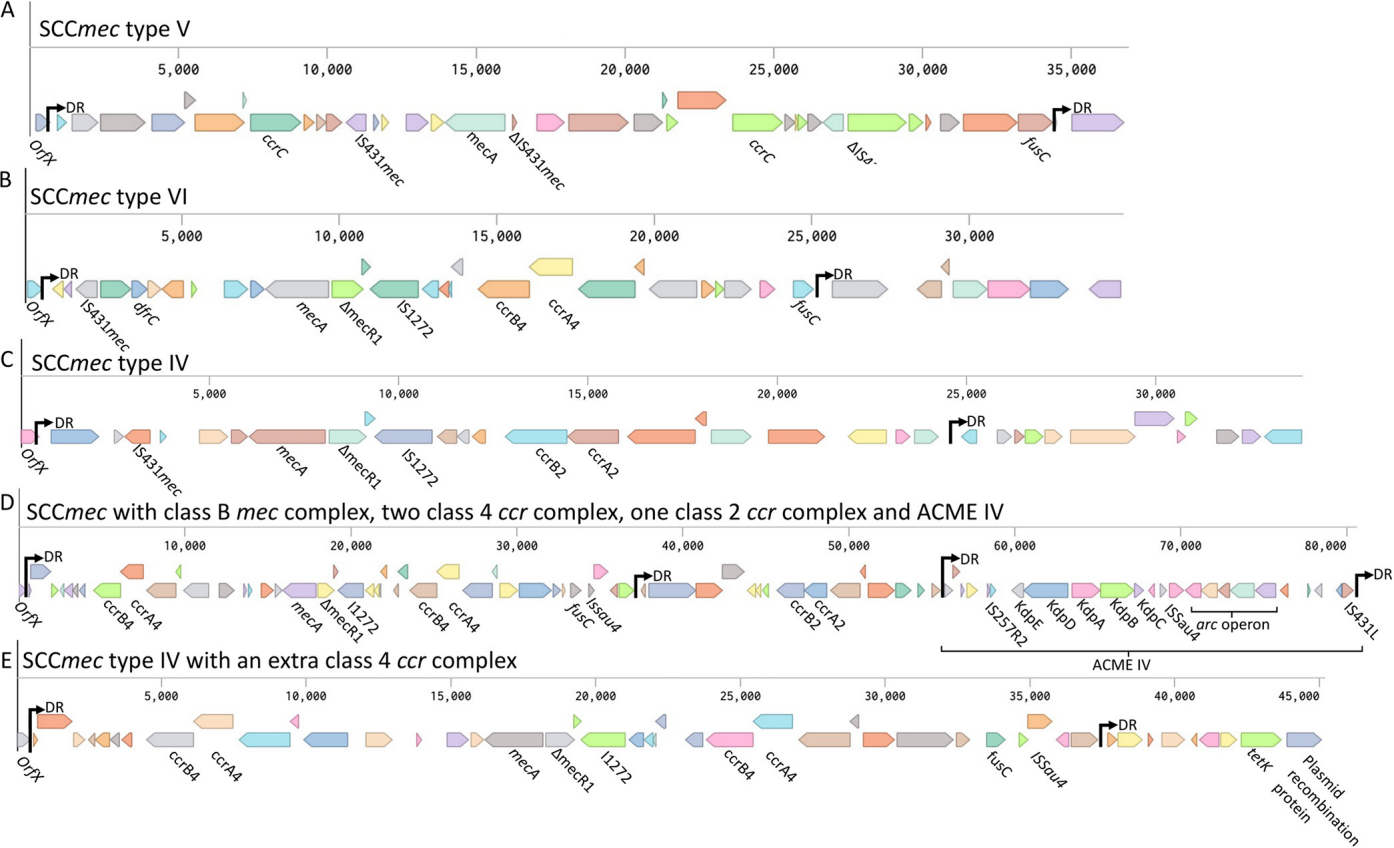
SCC*mec* structure found in the MRS isolates recovered from goats and their surrounding farm environments in Eastern Province, Saudi Arabia. (A) SCC*mec V* found in SA1, (B) SCC*mec* VI found in SA2 and SA4, (C) SCC*mec* VI found in SA3, SA5, SA6, SA7. (D) SCC*mec* with class B *mec* complex and two class 4, one class 2 *ccr* complexes and ACME IV element found in SE1. (E) SCC*mec* IV with an extra class 4 *ccr* complex found in SE2. DR = direct repeat sequence of the SCC*mec* attachment sites.

Seven out of nine MRS isolates sequenced (recovered from goats and the surrounding farm environments) were assigned to the SCC*mec* typing scheme (Fig. 6). However, we found that S. epidermidis isolates (Fig. 6D and E) possessed an additional *ccr* complex in their SCC*mec* elements and SE1 harbored the Arginine catabolic mobile element (ACME) IV. In SE1, the ACME, *ccr* class 2 and class 4 complexes were separated by direct repeat (DR) sequences. Moreover, we found that SCC*mec* V and SCC*mec* with class B *mec* complex and two class 4 and one class 2 *ccr* complexes carried the fusidic acid resistance gene *fusC* (Fig. 6A and D); the SCC*mec* VI (SA2 and SA4) carried *fusC* and the trimethoprim resistance gene *dfrC* (Fig. 6B) and SCC*mec* IV with an extra class 4 *ccr* complex (SE2) carried *fusC* and downstream of the second DR sequence adjacent to a plasmid recombination gene are the tetracycline resistance gene *tetK* (Fig. 6E).

## DISCUSSION

MRS can be transmitted across to an animal host from the environment and act as an intermediate for intraspecies and interspecies transmission. This study found that MRSA isolates recovered from goats were genetically similar to those isolated from their local farm environments. It is plausible to hypothesize that these MRSA isolates were transmitted to goats from the farm environment or vice versa. It is also plausible to suggest that CC5 MRSA transmission occurred between goats indirectly through an environmental intermediate based on the low SNP diversity and high gene correlation between goat and environmental isolates. Previous studies have shown that MRSA isolates were recovered from farm environments, including Danish minks and pig farms and Italian dairy cattle herds (22–24). These studies showed that the MRSA isolates recovered from the farm environments were similar to lineages associated with pigs (22–24). However, in this study, we found that the MRSA genotype ST5- SCC*mec* VI- t688 has been previously recovered from a patient in a hospital in Kuwait; ST6- SCC*mec* IV- t304 was genetically similar to that found in patients in hospitals in Oman and Egypt and the genotype ST5-SCC*mec* V-*spa* type t311 to that found in patients in a hospital in Italy but to the best of our knowledge unreported in livestock and companion animals (25–28). Therefore, it is most likely that these isolates were originally cross transmitted from humans to goats. This is unsurprising as there have been multiple reports of cross-transmission of the CC5 S. aureus lineage between humans, companion animals and livestock (29–32). Another indicator of human to animal transmission is the presence of the Panton-Valentine leukocidin toxin genes *lukF-PV* and *lukS-PV* found in SA3 and the staphylokinase gene *scn* and staphylococcal complement inhibitor gene *sak* found in all MRSA isolates, which are strongly associated with human infections and sporadically have been found in livestock (33–35). Furthermore, these isolates carried genetically similar plasmids found in S. aureus recovered from humans (including isolates from clinical samples, e.g., blood) and their environments in Europe, the USA, and Asia. However, we observed that the pSA1 plasmid found in ST5-MRSA-SCC*mec* VI- t688 genotype was also genetically similar to a plasmid (pLUH02) found in S. aureus ST5, which has been transmitted to poultry from humans, suggesting that this genotype may have further occurrence in other animals (31, 36). These isolates most likely have been transmitted to goats from local farmers based on the *fusC* gene found in four of the five SCC*mec* elements, as there was a high prevalence of these SCC*mec* genotypes in the Arabian gulf isolated from humans and livestock compared to other regions of the world (37–40).

We found that MRSE isolates recovered from goat nasal swabs possessing ST35 genotype have previously also been recovered from a patient in Iraq, shared bicycle in China, birds of prey in Portugal, associated with farmers and hospital-associated isolates in Belgium, clinical isolates in Portugal, environmental sources from Germany as well as isolates recorded on pubMLST (accessed 18 November 2021) from a human source in Germany, South Korea, and Russia (41–47). Another genotype identified in our study, ST153 has been previously reported in isolates recovered from samples of catheter-related bloodstream infections in Belgium, nasal swabs, subgingival sites and oral rinse in Ireland and an isolate recovered from human samples in Ireland, the USA, and Latvia (44, 48, 49). We, therefore, hypothesize that these two S. epidermidis sequence types are not regularly reported. However, these reports suggested that ST35 is shown to be a zoonotic isolate. Further surveillance of S. epidermidis in animals is required to understand whether these genotypes are truly zoonotic or whether the isolates were originally transmitted by farmers. In addition, we also identified high homology between the plasmids found in S. aureus and S. epidermidis isolates recovered from goats to those recovered from humans, further indicating that the source of MRSE isolates in goats may have been humans.

Phenotypic resistance to fluoroquinolones class of antibiotics was found in the MRS isolates recovered from goat and their farm environments. The correlation of fluoroquinolone resistance with MRS is not unusual, as reports have shown that the use of fluoroquinolones is a significant risk factor for MRSA isolation from patients, companion animals and livestock (50–54). Moreover, our findings that all MRS isolates were phenotypically resistant to trimethoprim-sulfamethoxazole, an antibiotic used for the MRSA decolonization and treatment, is worrying (55, 56). Reports of high rates of trimethoprim-sulfamethoxazole resistance within hospitals and communities are not uncommon; however, the prevalence of trimethoprim-sulfamethoxazole resistance in livestock is generally low and has been reported in only one study that 68.4% of the MRSA isolates recovered from swine in the South of Italy were trimethoprim-sulfamethoxazole resistance (57–59).

We found that the majority of the resistance phenotypes identified in all MRS isolates correlate with a known resistance genotype (*norA* gene for fluoroquinolones class of antibiotics, *mecA* for penicillin class of antibiotics, *LmrS* in all S. aureus and *dfrC* in all S. epidermidis for trimethoprim-sulfamethoxazole; *mgrA*, in S. aureus and S. epidermidis*; mepA*, *mepR* and *tet*(38) *in* S. aureus and *tet(K)* in SE2 for tetracycline class of antibiotics and *lmrS* in SA1 and SA7 and *mphC* and *msrA* in SE2 for erythromycin) (6, 60–66). Interestingly we found that the ST6-MRSA-SCC*mec* IV-t304 isolates (SA6 and SA7), recovered from goat nasal swab and soil samples were genetically identical except for one synonymous mutation. However, they were phenotypically different in their antibiotic resistance profile, as one isolate was resistant to erythromycin and tetracycline, whereas the other was sensitive to these antibiotics. We hypothesize that this may be due to the phenomenon known as “bias portioning” described in E. coli AcrAB-TolC multidrug efflux pump, which is distributed asymmetrically on the poles of the cell; when the cell divides, the mother cell inherits old poles that are phenotypically more effective at pumping out the antimicrobial drugs then the daughter cells (67). This may be similar to the multidrug efflux pumps *mgrA*, which can actively pump out the tetracycline class of antibiotics from the cell and *lmrS*, which can actively pump out the macrolides class antibiotics (erythromycin) from the cell. However, no experimental data have been reported to show that such a phenomenon occurs in staphylococci.

Finally, we found certain additional features in the SCC*mec* elements in MRS isolates recovered from goats that may pose further challenges in treating infections caused by these bacteria. This included two S. epidermidis isolates that did not fit into the standard SCC*mec* typing scheme due to having additional *ccr* complexes (68–70). Previous reports have shown that these SCC*mec* elements with additional *ccr* complexes can be isolated from clinical settings, communities, and human public environments (71–75). In addition, S. epidermidis isolates from bovine mastitis have been reported to carry a SCC*mec* IV element with an additional *ccr* class 4 complex similar to that found in our study (SE2) (76). Moreover, in this study, we identified a SCC*mec* element found in an MRSE isolate (SE1) that possessed 3 *ccr* complex (1 class 2 and 2 class 4 complexes) and the ACME IV element associated with increased ability to colonize the skin and mucosa, which was originally identified in a S. epidermidis ST153 isolate (49). The class 2 *ccr* complex and ACME IV element were separated by DR sequences attachment site of the SCC*mec* element in the host chromosome suggesting these genomic regions were acquired in separate horizontal transfer events (77). Based on previous studies, it is plausible that these adaptions found in such untypeable SCC*mec* elements may be advantageous for bacteria as multiple *ccr* complexes have increased susceptibilities to oxacillin or cefoxitin (72). Our analysis showed inclusion of multiple antibiotic resistance genes including *fusC* and *dfrC* (SA2 and SA4) on the same SCC*mec* element (SE1’s SCC*mec* IV+ *ccr4* class and SA2 and SA4’s SCC*mec* VI), which to the best of our knowledge, has not been reported previously. Furthermore, we found a possible inclusion of *fusC* and *tetK* (SE2 SCC*mec*) via the same SCC*mec* element; however, the integration of *tetK* within this element may have arisen separately via a recombinant plasmid as *tetK* was found adjacent to plasmid recombination protein. The inclusion of multiple antibiotic resistance genes on SCC*mec* elements may have a significant impact on public and animal health, making it more challenging to treat as well as increasing the potential of further dissemination of these antibiotic genes to other isolates via these mobile genetic elements.

The genetic similarities of MRS isolates recovered from goats and their farm environments indicate a possible transmission via the environment. Moreover, there is a strong indicator that these MRS isolates may have been initially transmitted from humans based on their molecular genotypes and plasmids possessed. These MRS isolates have also shown to be phenotypically resistant to multiple antibiotics, including trimethoprim-sulfamethoxazole used for decolonization patients with MRSA, which reduces options or treatment for animals and patients infected with these isolates. There were also isolates carrying two other antibiotic resistance genes apart from *mecA* (a SCC*mec* element with *fusC* and *tetK*, and another SCC*mec* element with fusC and *dfrC*) within the SCC*mec* element, which has the potential to transfer horizontally across to other staphylococci, making them multidrug resistant. Further large scale and structured surveillance studies are warranted to further our understanding of the human-livestock-environment cross transmission of these bacteria to improve hygiene practices in the farms.

## MATERIALS AND METHODS

### Sampling and bacterial isolation.

A total of 200 samples were collected from goats (nasal swabs; *n* = 130 and milk samples; *n* = 40) and their surrounding environments (soil; *n* = 15 and drinking water; *n* = 15) in one of the goat farms located in Eastern Province, Saudi Arabia, between November 2019 to August 2020. Nasal swabs were collected from nostrils after thorough cleaning and disinfection of the external nares. The collected swabs were kept in Amie’s transport medium (Difco, BD, Franklin Lakes, NJ, USA) and transported to the laboratory for microbiological examination. Milk samples were collected from lactating does in a sterile screw-cap tube, following the standard methods described by the National Mastitis Council (NMC, 1990). Environmental samples (soil and drinking water) were collected from herds in a sterile screw-cap container. All samples were labeled and transported cooled to the laboratory in an icebox (4°C). Samples were plated onto blood agar, Baird–Parker agar (Oxoid, Basingstoke, Hampshire, UK) supplemented with egg yolk tellurite and mannitol salt agar (Oxoid, Basingstoke, Hampshire, UK) and then incubated aerobically at 37°C for 24 h. A single morphologically typical staphylococcal isolate per sample was purified on 5% sheep blood agar (Oxoid, Basingstoke, Hampshire, UK) and further characterized by Gram staining, coagulase, and catalase test. Identification of staphylococci isolates were further confirmed to the species level by Vitek 2 COMPACT system (bioMérieux, Marcy l'Etoile, France) using Gram-positive cards, following the manufacturer guidelines.

### Antibiotic susceptibility testing.

The phenotypic resistance profiles of all staphylococci isolates were determined by an automated Vitek 2 COMPACT system using AST-GP67 card (bioMérieux, Marcy l'Etoile, France), following the manufacturer guidelines. MIC values were interpreted according to the recommendations of EUCAST, 2021(78).

### Whole genome sequencing and genomic assembly.

Nine isolates that showed resistance to methicillin were selected and submitted for whole genome sequencing (WGS) by MicrobeNG (Birmingham, UK) using Illumina sequencing platforms (San Diego, CA, USA). Short read files were deposited in European Nucleotide Archives (ENA) under the study PRJEB49547. The accession numbers for each isolate are included in the supplementary data (Table S1).

The quality of reads from sequencing was analyzed using the fastQC software, and reads were trimmed using the Trimmomatic software by setting the phred cutoff to Q20 (79, 80). Genomes and plasmids were assembled from the trimmed paired-end reads using SPades 3.15.3 (81).

### Genome annotation and genetic typing.

Genomes and plasmids were initially annotated using Prokka 1.14.6 and then further annotated for antibiotic resistance genes/mutations using the Comprehensive Antibiotic Resistance Database; acquired virulence genes using the VirulenceFinder 2.0 webserver and insertion sequence using ISfinder webserver (82–85).

For genetic typing isolates, the python script mlst was used (https://github.com/tseemann/mlst) with the most up to date database obtained from pubMLST (accessed July 2021) to determine their multilocus sequence type (MLST); the SCC*mec*Finder 1.2 webserver for typing isolate SCC*mec* elements and spaTyper 1.0 webserver to type S. aureus spa gene (86, 87). SCC*mec* attachment sites were identified by blast searching against a list of known attachment sites direct repeats (DR) (88).

### Genomic and plasmid comparison.

A digital DNA-DNA hybridization (dDDH) analysis using the TYGS webserver was conducted to determine the overall similarities between the genomes (89). To determine the pairwise SNP distance of the core genomes between the isolates, the Parsnp 1.5.6 alignment tool was employed using the reference genome DSM 20231 (accession no.: CP011526.1) for S. aureus and the reference genome ATCC 144990 (accession no.: CP035288.1) for S. epidermidis isolates. In addition, the analysis enabled recombination filtered, and the snp-dists tool (https://github.com/tseemann/snp-dists) was used to convert alignment to SNP distance matrix (90). For gene correlation, the pangenome pipeline roary was used to group genes that have a blastp minimum of 90% and the r package “corr” (https://cran.r-project.org/web/packages/corrr/index.html) to determine their Pearson correlation coefficient (91).

The plasmid novelty was determined using the mash dist function on the PLSDB webserver (92). A blast comparison of plasmids that had hits of similarities was conducted and visualized using the CGviewer webserver (93).

### Hierarchy clustered heatmap.

Hierarchy clustered heatmap for genomic comparison was constructed using the r package “pheatmap.”

### Ethical approval.

All experimental procedures used in the current study were approved by the guidelines of the Ethics Committee at King Faisal University, Saudi Arabia (Approval no: DSR-691).
